# Refining a major QTL controlling spotted wilt disease resistance in cultivated peanut (*Arachis hypogaea* L.) and evaluating its contribution to the resistance variations in peanut germplasm

**DOI:** 10.1186/s12863-018-0601-3

**Published:** 2018-03-23

**Authors:** Zifan Zhao, Yu-Chien Tseng, Ze Peng, Yolanda Lopez, Charles Y. Chen, Barry L. Tillman, Phat Dang, Jianping Wang

**Affiliations:** 1 Agronomy Department, University of Florida, Gainesville, FL 32610 USA; 2 North Florida Research and Education Center, University of Florida, Marianna, FL 32446 USA; 3 Department of Crop, Soil and Environmental Sciences, Auburn University, Auburn, AL 36849 USA; 4 USDA-ARS National Peanut Research Laboratory, Dawson, GA 39842 USA; 5 Center for Genomics and Biotechnology, Key Laboratory of Genetics, Breeding and Multiple Utilization of Crops, Ministry of Education; Fujian Provincial Key Laboratory of Haixia Applied Plant Systems Biology, Fujian Agriculture and Forestry University, Fuzhou, Fujian 350002 China

**Keywords:** Cultivated peanut, Tomato spotted wilt virus (TSWV), Spotted wilt, Simple sequence repeat (SSR), Single nucleotide polymorphism (SNP), Spotted wilt disease, Quantitative trait loci (QTL), US peanut mini-core germplasm

## Abstract

**Background:**

Spotted wilt, caused by *tomato spotted wilt virus* (TSWV), has been one of major diseases in cultivated peanut grown in the southeastern United States (US) since 1990. Previously a major quantitative trait locus (QTL) controlling spotted wilt disease resistance was mapped to an interval of 2.55 cM genetic distance corresponding to a physical distance of 14.4 Mb on chromosome A01 of peanut by using a segregating F_2_ population. The current study focuses on refining this major QTL region and evaluating its contributions in the US peanut mini-core germplasm.

**Results:**

Two simple sequence repeat (SSR) markers associated with the major QTL were used to genotype F_5_ individuals, and 25 heterozygous individuals were selected and developed into an F_6_ segregating population. Based on visual evaluation in the field, a total of 194 susceptible F_6_ individuals were selected and planted into F_7_ generation for phenotyping. Nine SSR markers were used to genotype the 194 F_6_ individuals, and QTL analysis revealed that a confidence interval of 15.2 Mb region had the QTL with 22.8% phenotypic variation explained (PVE). This QTL interval was further genotyped using the Amplicon-seq method. A total of 81 non-redundant single nucleotide polymorphism (SNP) and eight InDel markers were detected. No recombinant was detected among the F_6_ individuals. Two InDel markers were integrated into the linkage group and helped to refine the confidence interval of this QTL into a 0.8 Mb region. To test the QTL contributes to the resistance variance in US peanut mini-core germplasm, two flanking SSR markers were used to genotype 107 mini-core germplasm accessions. No statistically significant association was observed between the genotype at the QTL region and spotted wilt resistance in the mini-core germplasm, which indicated that the resistance allelic region at this QTL didn’t contribute to the resistance variance in the US peanut mini-core germplasm, thus was a unique resistance source.

**Conclusion:**

A major QTL related to spotted wilt disease resistance in peanut was refined to a 0.8 Mb region on A01 chromosome, which didn’t relate to spotted wilt disease resistance in the US peanut mini-core germplasm and might be a unique genetic source.

**Electronic supplementary material:**

The online version of this article (10.1186/s12863-018-0601-3) contains supplementary material, which is available to authorized users.

## Background

Peanut or groundnut (*Arachis. Hypogaea* L.) is one of the most important oilseed crops planted all over the world [1]. It is an annual herbaceous plant belonging to the botanical family Fabaceae and usually grown in tropical, subtropical or warm-temperature regions with moderate rainfall. Peanut originates from South America, with natural distribution restricted to Brazil, Bolivia, Paraguay, Argentina, and Uruguay [2]. Global peanut production fluctuates between 31 and 35 million metric tons per year (International Nut and Dried Fruit Foundation, http://www.nutfruit.org), and it is mainly consumed as a great source of protein and oil worldwide. As an important economic crop, peanut production is threatened by many diseases.

Spotted wilt, caused by *tomato spotted wilt virus* (TSWV), was first observed in Texas, USA in 1971 [3] and has become increasingly epidemic in peanut and other crop production systems in the Southern US since 1985 [4]. Peanut plants infected by TSWV show stunting, specifically when TSWV infects the plant at an early developmental stage. Besides stunting, infected leaves usually have chlorosis, necrosis or ring spots [5]. It was estimated that 50% of the peanut crop grown in southern Texas was lost due to spotted wilt in 1985 [6] and peanut yield losses progressively increased from the late 1980s to 1997.

The current paradigm for combatting spotted wilt in peanut is through the selection of resistant cultivars. However, the severity of spotted wilt disease can vary dramatically from year to year depending on the environment [7, 8]. The incidence of asymptomatic infection can be high [9], which makes phenotypic selection of resistant plant in the field difficult. The use of molecular markers is one of the primary efforts to develop improved disease resistant cultivars in breeding programs. Comparing to visual selection, marker assisted selection (MAS) has been shown to increase the genetic gain significantly per selection cycle [10]. To realize MAS for spotted wilt resistant cultivar development, it is essential to identify the makers closely linked to the spotted wilt resistance.

Cultivated peanut is a tetraploid (2n = 4× = 40, AABB) with two different sets of chromosomes from A and B genomes of two different progenitor species [11]. The genome size of cultivated peanut is approximately 2.7 Gb [12]. The pace of developing genetic markers in cultivated peanut was relatively slow in the past, which was mainly due to the complexity of peanut tetraploid genomes and low levels of detected polymorphism [13]. However, current advancement of next generation sequencing (NGS) technologies has greatly facilitated the development of molecular markers, especially through the use of simple sequence repeats (SSRs) and single nucleotide polymorphisms (SNPs). In current plant genetic research and breeding programs, SSRs and SNPs are much more favored than other types of markers due to their abundancy, co-dominancy, and highly repeatability [14, 15]. Many molecular markers have been reported to be linked with loci controlling resistance to diseases.

For spotted wilt resistance in cultivated peanut, quantitative trait loci (QTLs) were reported by Guo’s group (USDA ARS, Tifton, GA) with two populations at different generations [16–19]. One population was derived from a cross between SunOleic 97R and NC 94022, so called S population, and the other population was derived from a cross between Tifrunner and GT-C20, so called T population. Based on the latest report on the S population, six QTLs related to spotted wilt resistance with PVE ranging from 4.36% to 29.14% were identified, among which five QTLs were found to be located on the A01 while the other one was on A09 [18]. For the T population, 11 spotted wilt-related QTLs were identified with PVE ranging from 6.74% to 14.41% [19]. Recently, in an F_2_ population derived from a cross between Georgia Valencia and Florida-EP™ ‘113’, one major QTL with PVE around 22.7% was identified on the A01 chromosome [20]. So far, no closely linked markers have been developed and utilized for MAS of spotted wilt resistance in peanut breeding programs most likely due to lack of concordance between multiple QTLs identified in different populations and different years. This study was intended to validate and refine a major QTL related to spotted wilt resistance to identify markers closely linked to TSWV resistance that would have utility in peanut breeding programs.

Germplasm collections are excellent sources for peanut breeders to broaden the genetic basis of the breeding materials and to incorporate the important alleles associated with valuable traits. The US mini-core germplasm collection representing the whole US peanut germplasm collection has been evaluated for different traits, such as resistance against different diseases [21] including resistance to TSWV infection. But it is largely unknown whether the QTL of our interest contributes to the spotted wilt resistance of the accessions in (or in another word, exist in the gene pool of) the US mini-core germplasm collection.

The objectives of this study were to refine the major QTL related to the spotted wilt resistance contributed by Florida-EP™ ‘113’ in cultivated peanut and survey its prevalence in the US peanut mini-core germplasm collection. The refined genome position of this QTL will provide useful information for developing specific and effective markers for MAS of spotted wilt resistance in peanut breeding programs.

## Methods

### Plant material

A cross between Florida-EP™ ‘113’ and Georgia Valencia was initiated in 2009 at the North Florida Research and Education Center (NFREC) near Marianna, FL. Florida-EP™ ‘113’ is a new runner type variety developed by the University of Florida (UF) peanut breeding program with high resistance to TSWV [22]. Georgia Valencia is a valencia type variety developed by the Georgia Agricultural Experiment Station and is highly susceptible to spotted wilt [23]. The Florida-EP™ ‘113’ was derived from a cross between NC94022 and ANorden [24], and NC94022 had very high resistance to spotted wilt. From F_2_ to F_5_ generations, seeds were bulk harvested without selection. In the F_5_ generation, a subset of 245 individuals was genotyped using two SSR markers on chromosome A01, which are GM 1694 and ARS 721 [20]. Individuals showing the heterozygous genotype at both marker loci were selected and planted to generate the F_6_ families at NFREC in 2015. Visually susceptible F_6_ plants were selected for DNA extraction and their seeds were harvested individually and planted to form the F_7_ generation in 2016 for phenotyping.

For the US peanut mini-core germplasm accessions, seeds of 107 accessions in the collection were planted with four replicates in a completely random design in 2012 at Plant Science Research and Education Unit (PSREU) near Citra, Florida. These accessions were again planted at North Florida Research and Education Center (NFREC) in April 2016 with two replicates in a randomized complete block design.

### Phenotyping with visual rating and ELISA test

Each visually susceptible F_6_ plant was individually harvested and planted into a whole plot in the F_7_ for phenotyping with one plot corresponding to one susceptible plant in the F_6_ generation. Both visual rating and ELISA tests were used to phenotype the F_7_ lines. Plots were 0.9 m in width and 4.5 m in length, containing two rows with planting density one plant per 0.3 m. Visual rating was used to evaluate the infection severity of one whole plot before the plot was harvested. A scale of 1 to 10 was used to represent the percentage of infected plants (1, 2, 3, 4, 5, 6, 7, 8, 9, 10 equals 1–10%, 11–20%, 21–30%, 31–40%, 41–50%, 51–60%, 61–70%, 71–80%, 81–90%, and 91–100% respectively) [20].

The ELISA test was conducted with the TSWV PathoScreen Kit (Agdia, Inc., IN). For sample preparation, a maximum 10 roots from each plot were randomly collected, dried and stored in paper bags. Lateral roots in each sample were removed and the root crown was crushed with a hammer. A 0.1 g sub-sample of this crushed root crown sample was put into a 2 mL tube and grounded using a Mixer Mill Grinder (Retsch Inc, PA) at 30 times s^− 1^ for 1.5 min. General extract buffer of 1 mL was added into each tube after grinding and incubated overnight at 4 °C. These steps were followed according to the PathoScreen Kit User Guide. Root crown samples from spotted wilt-resistant parent Florida-EP™‘113’ were used as the negative control. The percentage of infected roots in all roots tested from one plot was calculated as infection rate for the plot.

The US peanut mini-core germplasm accessions were phenotyped using the visual rating method in both 2012 and 2016 just prior to harvesting, and the same scale and rule were followed as described above. The mini-core germplasm accessions were also mechanically inoculated in greenhouse and phenotyped by ELISA. Specifically, the accessions were grown in the greenhouse at a temperature of 25 to 30°C, and 60 to 90% relative humidity. Nine seeds per accession were sown in plastic seedling trays (7.87 cm × 7.87 cm × 5.92 cm per cell) containing all-purpose professional growing mix consisting of Canadian sphagnum peat moss, coarse perlite, vermiculite, and dolomitic limestone (Sun Gro Horticulture, Agawam, MA). Peanut plants at two- to three-leaf stage (7 to 9 days after planting [DAP]) were dusted with carborundum, and the TSWV inoculum (1 ml per plant) was applied by rubbing both surfaces of the leaf with a cotton swab. After inoculation, the sap and carborundum were rinsed from the seedlings with distilled water and the plants were kept in the greenhouse under the same environmental conditions as previously mentioned. Inoculated plants were observed daily for symptom development. Plants were considered to have localized infection when chlorotic rings or concentric rings developed only on the inoculated leaves, and without any symptoms on new leaves. The plants were considered to be systemically infected when the symptoms developed on new emerging leaves. The plants were monitored in the greenhouse for 40 days after inoculation. The percentage of infected plants was recorded at 40 days post inoculation (DPI). At 40 DPI, 0.2 g of roots was collected from every plant to assay by ELISA test using TSWV-specific antiserum (Agdia Inc, IN).

### Genotyping with SSR markers

Genomic DNA was extracted using cetyltrimethyl ammonium bromide (CTAB) method. DNA quality and concentration was checked using 1% agarose gel and Quant-iT™ Picogreen dsDNA Assay Kit. Extracted DNA was diluted to 10 ng/μL for polymerase chain reaction (PCR) with SSR markers. PCR was done in 10 uL volume including 1 μL of 10 × PCR buffer, 1.25 μL of Magnesium Chloride (25 mM), 1 μL of dNTP (2 mM), 0.25 μL of Taq enzyme, 2 μL of forward and reverse primers (2 mM), 2 μL of DNA template (10 ng/μL), and 2.5 μL of distilled deionized water. The PCR was operated using a touchdown program with an initial denaturation at 95°C for 4 min; 10 cycles of amplification at 95°C for 30 s, 65°C for 30 s, 72°C for 1 min; 30 cycles of amplification at 95°C for 30 s, 55°C for 30 s, 72°C for 1 min; and a final extension at 72°C for 7 min. PCR products were separated using 6% polyacrylamide gel electrophoresis (PAGE) under 150 V for 2 h in 1X TBE buffer. The gels were stained with Ethidium Bromide (EB) for 10 mins before visualizing under UV light.

### Data analysis

R (version 3.3.3) was used to conduct the ANOVA test and Tukey’s Honest Significant Test (TukeyHSD) on the phenotypic data sets. For phenotypic data from the mapping population, a one-way ANOVA test was conducted at significance level of *P* < 0.05. For phenotypic data from the mini-core germplasm, one-way ANOVA (RCBD) test was conducted to check whether the visual rating result was significant between resistant and susceptible genotypes, and was used for visual rating data in 2012 and 2016 separately because the visual rating was taken in two different places. Results were regarded as significant if *P* < 0.05. A two sample t-test was used to check whether the ELISA result was significant between resistant and susceptible genotypes.

### Amplicon-seq

Amplicon-seq was used to develop SNP markers in the interval of interest. Primers were designed to be evenly distributed across this interval based on the diploid wild peanut genome sequence [12]; however, some specific regions with highly repetitive DNA or missing sequence information were avoided. Primer sequences were mapped to the genome reference by using Bowtie (−f –v 2 –I 100 –X 8000) [25]. Primers with multiple hits across the genome were eliminated. The length of each amplicon was approximately 7 kb. KOD Xtreme Hot Start DNA Polymerase (EMD Millipore, MA), which is an optimized PCR enzyme for the amplification of long DNA templates, was used for PCR reaction. The PCR was operated using a 2-step cycling with polymerase activation at 94°C for 2 min; 30 cycles of denaturation at 98°C for 10 s and annealing and extension at 68°C for 1 min. A total of eight samples were amplified and the PCR products were checked on 1% agarose gel. Subsequently, successfully-amplified amplicons from each sample were equally pooled and submitted to the Interdisciplinary Center for Biotechnology Research (ICBR) at the University of Florida for product-cleaning and library construction. Illumina MiSeq SE 1 × 300 was used for amplicon pool sequencing.

Raw sequences were trimmed with Trimmomatic [26] and the quality of trimmed data was checked by using FastQC [27]. The clean sequence reads were aligned to diploid peanut genomes [12] using aligner BWA MEM [28]. After alignment, SNP calling was conducted with three different software: GATK [29], freebayes [30] and Samtools [31] using parameters previously described [32]. Results from these three SNP callers were compared and genotyping results from newly-detected SNPs at the targeting interval were summarized.

### Construction of linkage map and QTL analysis

QTL IciMapping v3.3 [33] was used for both linkage map construction with MAP function and QTL analysis with BIP function. For linkage map construction, the logarithm of odds (LOD) was set at 3 for grouping, and the nearest neighbor combined with two-opt algorithm (nnTwoOpt) was implemented as the algorithm for ordering. Sum of Adjacent Recombination Frequencies (SARF) was used for rippling with window size of five markers. For QTL analysis, inclusive composite interval mapping (ICIM) function was applied with additive method, using 1 cM as step and 0.001 probability in the stepwise regression. Individuals with less confident phenotype (number of tested roots for ELISA was less than 4)and with extreme influence on the QTL result were filtered out. The QTL results with LOD score above 3 was regarded as significant.

## Results

### Mapping population

Of 245 F_5_ individuals genotyped with the two SSR markers, GM 1694 and ARS 721, 24 plants were heterozygous genotype for both marker loci. The seeds of these 24 plants were harvested individually and planted into F_6_ families comprised of a total 2200 individual plants. We visually selected 194 F_6_ plants, which showed spotted wilt disease symptoms to form the mapping population for genotyping and phenotyping.

### Phenotyping TSWV resistance of the mapping population

**Fig. 1:**
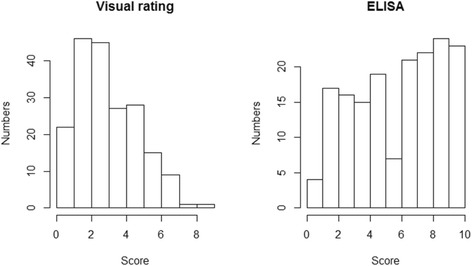
Visual rating of spotted wilt disease and enzyme linked immunosorbent assay (ELISA) test of tomato spotted wilt virus. For visual rating, the score (X axis) was based on the percentage of infected plants in a whole plot while the score for ELISA (X axis) was based on the percentage of infected root samples in all tested root samples that were randomly collected from each plot. Y axis shows the plot number of a given score

A total of 194 F_7_ plots were evaluated for spotted wilt severity by visual rating and ELISA. The score of all plots from visual rating ranged from 0 to 9, with an average score of 3.4, while the score of all root samples from ELISA ranged from 0 to 10, with an average score of 5.7 (Fig. 1). Sixteen root samples from Florida-EP™‘113’ were included in the ELISA and none of these tested positive for infection, which confirmed the high level of TSWV resistance of this variety.

### Genotyping the mapping population

**Fig. 2:**
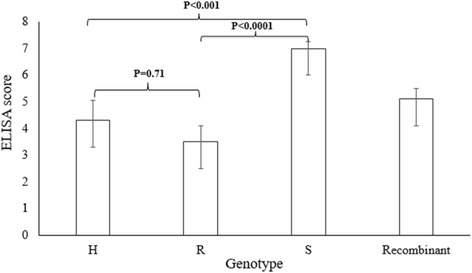
Comparison between the enzyme linked immunosorbent assay (ELISA) results from different genotype groups. The average ELISA scores of heterozygous (H), resistant (R), susceptible (S) and recombinant genotype groups are 4.23, 3.54, 7.05 and 5.05 respectively. All data are given as means±standard deviation of the mean (s.d.m)

A total of nine SSR markers were used to genotype the fine mapping population. Among 194 individuals genotyped, 20 individuals had the same genotypes at all nine loci as the Florida-EP™ ‘113’, the TSWV resistant parent, while 103 individuals had the same genotypes at all nine loci as the Georgia Valencia, the spotted wilt susceptible parent, and 16 individuals were heterozygous at the nine loci, with the remaining 55 individuals being recombinants (Additional file 1). Therefore, more than half of this population was basically fixed for the susceptible genotype at the target region on the A01 chromosome. This result corresponded to the phenotypic selection based on TSWV susceptibility in the F_6_ generation. The average ELISA score for the resistant genotype was 3.54, while the average score for the susceptible genotype was 7.05 (Fig. 2). In terms of heterozygous and recombinant individuals, the average scores were 4.23 and 5.05, respectively (Fig. 2). The overall phenotypic difference among the four genotype groups was significant based on ANOVA test (*P* < 0.0001). However, no statistical difference was observed between the ELISA scores of heterozygous and resistant genotype groups (*P* = 0.71), while the ELISA scores of resistant and heterozygous genotype groups was lower than the susceptible genotypes (resistant vs susceptible genotype groups: *P* < 0.0001; heterozygous vs susceptible genotype groups: *P* < 0.001).

### QTL analysis based on the mapping population

**Fig. 3:**
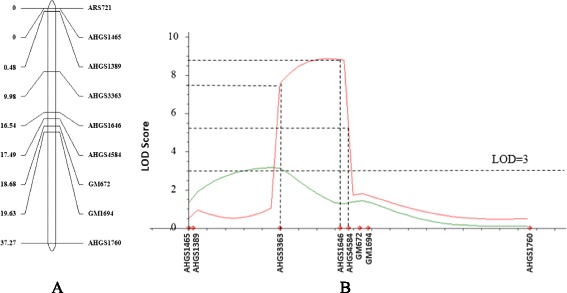
Linkage map (**a**) and quantitative trait locus (QTL) analysis (**b**) with nine simple sequence repeat (SSR) markers. The total length of linkage map is 37.27 cM, with two markers located at the same position. For the QTL analysis, phenotyping results from both visual rating and enzyme linked immunosorbent assay (ELISA) test are included, and represented using green and red lines respectively

A linkage map of the 37.27 cM target region was generated with the mapping population genotyped with nine SSR markers (Fig. 3). SSR markers, ARS721 and AHGS1465 showed exact the same genotyping result and, thus were mapped to the same location although there were 1.2 Mb physical distance between the two markers. QTL analysis based on visual ratings revealed one QTL with a LOD score of 3.15 at the marker AHGS 3363 (at 42.6 Mb position on chromosome A01) with PVE of 7.7%. QTL analysis using ELISA detected a QTL region flanked by markers AHGS 3363 (at 42.6 Mb position) and AHGS 1646 (at 43.4 Mb position) with a LOD score 8.87 and PVE of 22.8%. The confidence interval for the QTL based on ELISA was between AHGS 3363 and AHGS 4584 with a physical distance of 15.2 Mb.

### Amplicon-seq to develop markers within the new interval

**Fig. 4:**
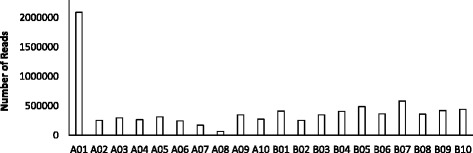
Distribution of unique aligned reads across the A and B reference genomes. About 25% of the total unique aligned reads can be aligned to A01 chromosome

**Fig. 5:**
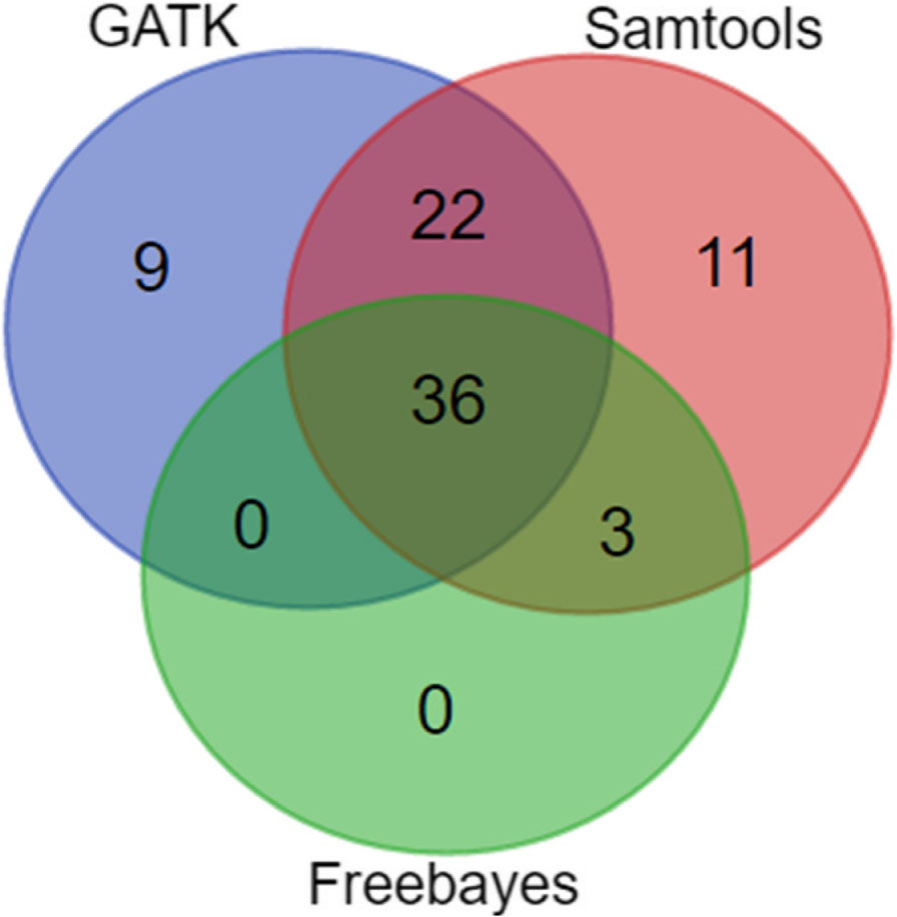
Venn diagram showing single nucleotide polymorphism (SNP) calling results from three software GATK, Samtools and Freebayes. A total of 81 non-redundant SNPs were called by software, among which 36 were common SNPs

**Fig. 6:**
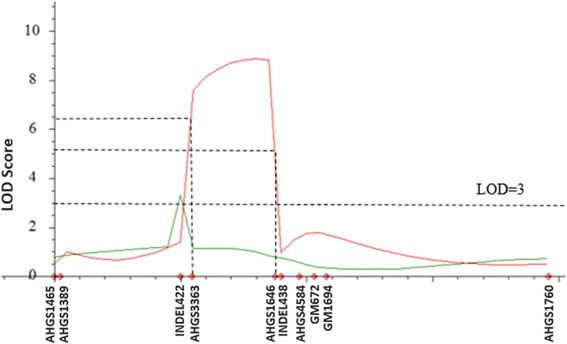
Quantitative trait locus (QTL) analysis of the targeting region in peanut A01 chromosome in the whole population. Phenotyping results from both visual rating and enzyme linked immunosorbent assay (ELISA) test are included, and represented using green and red lines respectively. One QTL was detected in both QTL analysis with the Logarithm of Odds score threshold 3

A total of 104 pairs of primers were designed across the QTL interval within the genomic region from 42 to 44.5 Mb on the A01 chromosome. Eight samples were amplified by the 104 pairs of primers and the amplicons were sequenced. The eight samples included the two parental cultivars (Georgia Valencia and Florida-EP™‘113’), one resistant F_6_ individual (1082/12 m), one F_6_ susceptible genotype with three susceptible individuals’ DNA pooled together, two heterozygous genotypes (1082/25 m and 1075/8 m) and one recombinant genotype (1082/10 m). Overall, the amplicon-seq generated 14,516,976 raw reads from the Illumina sequencing. After trimming, 98.18% of reads survived and 93.76% of reads could be aligned to peanut A and B genomes, with 58.65% of reads uniquely aligned (Table 1). Among all uniquely aligned reads, 25% of them could be aligned to the A01 chromosome, with the rest of the uniquely aligned reads aligned to other chromosomes, contigs and scaffolds in the peanut reference genomes (Fig. 4). A total of 81 non-redundant SNPs were called on the A01 chromosome between two parental lines, and 36 of them were common SNPs from three SNP calling software packages, Samtools, GATK and freebayes (Fig. 5). Among all eight samples sequenced, the SNP genotyping results were highly consistent with the SSR genotypes harboring the target region (Table 2). Three InDels with sequence length variation equal to or above two bases were detected with Samtools. Two InDels were successfully applied to genotype the whole population (InDel422 and InDel438). Both InDels were included in the linkage map and QTL analysis, which helped to refine the QTL region within a 0.8 Mb interval (Fig. 6). A total of nine gene models were located in the QTL interval (Table 3) and none of them were annotated as resistance genes.

**Table 1:** Statistics for the Amplicon-seq

Sample	Amplicons (% of total amplicons)	Raw Reads	Clean Reads(% of raw reads)	Alignment to A + B(% of raw reads)	Unique aligned to A + B(% of raw reads)
GV	85(19.45%)	2,239,584	2,198,691(98.17%)	2,138,865(95.50%)	1,414,454(63.16%)
FL-113	64(14.65%)	1,968,429	1,936,715(98.39%)	1,886,187(95.82%)	1,175,212(59.70%)
1082/12 m	37(8.50%)	1,577,182	1,542,703(97.81%)	1,472,688(93.37%	894,211(56.70%)
1082/25 m	52(11.90%)	1,530,410	1,498,663(97.93%)	1,417,204(92.60%)	846,556(55.32%)
S Pool	53(12.13%)	1,651,158	1,601,860(97.01%)	1,507,515(91.3%)	949,213(57.49%)
1082/10 m	52(11.90%)	1,802,447	1,762,463(97.78%)	1,670,078(92.66%)	1,035,643(57.46%)
1075/1 m	44(10.07%)	1,766,476	1,749,951(99.06%)	1,661,645(94.07%)	1,030,698(58.35%)
1075/8 m	50(11.44%)	1,981,290	1,961,693(99.01%)	1,857,054(93.73%)	1,153,221(58.21%)
Total	437	14,516,976	14,252,739(98.18%)	13,611,236(93.76%)	8,513,628(58.65%)

S Pool = Pool of DNAs from three phenotypically susceptible plants; A + B = A and B reference genome of *Arachis duranensis* and *Arachis ipaensis* from Bertioli et al., 2016

**Table 2:** Single nucleotide polymorphism markers detected using Amplicon-seq between two single sequence repeat markers

SNP ID:	Position	GV	FL-113	1082/12 m	1082/25 m	S Pool	1082/10 m	1075/1 m	1075/8 m
AHGS3363(SSR)	42,634,085	S	R	R	H	S	R	H	H
Aradu.A0142687154.CT	42,687,154	S	R	.	H	.	R	.	.
Aradu.A0142689470.GA	42,689,470	S	R	.	H	S	R	.	.
Aradu.A0142693606.CA	42,693,606	S	R	.	H	S	R	.	.
Aradu.A0142787243.GT	42,787,243	S	R	R	H	S	H	H	H
Aradu.A0142809398.CT	42,809,398	S	R	.	.	.	H	H	H
Aradu.A0142877078.GA	42,877,078	S	R	R	H	S	.	H	H
Aradu.A0142879480.CT	42,879,480	S	R	R	H	S	H	H	H
Aradu.A0142911160.CT	42,911,160	S	R	R	.	.	H	.	H
Aradu.A0142913756.CT	42,913,756	S	R	R	.	.	H	H	H
Aradu.A0142929460.GA	42,929,460	S	R	.	H	S	H	H	H
Aradu.A0143065764.CT	43,065,764	S	R	R	.	.	.	.	H
AHGS1646(SSR)	43,349,531	S	R	R	H	S	H	H	H

*SNP* single nucleotide polymorphism, *SSR* single sequence repeat, *S Pool* Pool of DNAs from three phenotypically susceptible plants; “.” indicates missing data

**Table 3:** Gene models in the refined quantitative trait locus interval

Gene ID	chromosome	Start (bp)	End (bp)	Cover(Y/N)	annotation
Aradu.30S8W	Aradu.A01	42,645,388	42,650,347	N	NAC domain protein
Aradu.FSC6M	Aradu.A01	42,712,369	42,718,645	N	vacuolar-processing enzyme-like proteolysis
Aradu.5G7S4	Aradu.A01	42,738,234	42,741,762	N	NADH dehydrogenase (ubiquinone) complex
Aradu.73KGG	Aradu.A01	42,895,785	42,899,760	Y	heat shock protein STI-like isoform
Aradu.1I2B8	Aradu.A01	42,900,430	42,904,100	Y	elongation factor Tu GTP-binding domain protein
Aradu.P5RSR	Aradu.A01	42,916,210	42,918,580	Y	uncharacterized protein
Aradu.J20LR	Aradu.A01	42,972,238	42,973,190	Y	Uncharacterized protein
Aradu.VHJ4V	Aradu.A01	43,191,793	43,194,358	N	SNARE associated Golgi protein
Aradu.L0WTK	Aradu.A01	43,351,230	43,356,187	N	DDB1-binding WD40 protein

Y/N=Yes/ No, which indicates whether the gene model was covered by Amplicon-seq or not

### Evaluating the contribution of the QTL identified above to the resistance variations in the US peanut mini-core germplasm

A total of three phenotyping datasets were collected from the US peanut mini-core germplasm, including visual rating data in 2012, and in 2016 in Florida and the ELISA results from Auburn University in 2016. The average scores for the visual data collected in 2012, 2016, along with the ELISA phenotyping results were 4.09, 4.31, and 5.21, respectively.

Two SSR markers AHGS 3363 and AHGS 1646, which harbored the major QTL related to spotted wilt resistance identified above, were used to genotype the 107 US peanut mini-core germplasm accessions. Because of the wide genetic diversity of the US peanut mini-core germplasm, the 107 mini-core accessions showed multiple band patterns at both SSR loci with six and eight band patterns at AHGS 3363 and AHGS 1646, respectively. For AHGS 3363, 20 accessions showed the band pattern of susceptible Georgia Valencia and 22 accessions showed the band pattern of resistant Florida-EP™‘113’. At the AHGS 1646 locus, 12 and 27 accessions showed susceptible and resistant band patterns, respectively. Only two and one showed the susceptible and resistance band pattern at both marker loci, respectively.

Phenotypic results between the accession groups showing susceptible and resistant band patterns in the US peanut mini-core germplasm based on the two SSR markers were compared. At marker locus AHGS 1646, the averages of visual ratings in 2012, 2016, and the ELISA of the accession groups showing susceptible and resistant band patterns were 4.14 vs 4.52, 4.15 vs 4.31, and 5.5 vs 5.45, respectively. At marker AHGS 3363, they were 4.44 vs 3.92, 4.49 vs 3.99, and 4.58 vs 5.25, respectively. No statistically significant differences were observed between the susceptible and resistance genotypes at either of the SSR locus (Table 4).

**Table 4:** ANOVA test results of two genotypes at two single sequence repeat loci using three different datasets in the US peanut mini-core germplasm

SSR Marker	AHGS 1646			AHGS 3363		
Genotype(S/R)	S	R	*P*	S	R	*P*
2012 Visual	4.14	4.52	0.54	4.44	3.92	0.13
2016 Visual	4.15	4.31	0.73	4.49	3.99	0.18
ELISA	5.5	5.45	0.94	4.58	5.25	0.48

*S* band pattern following spotted wilt susceptible cultivar Georgia Valencia, *R* band pattern following spotted wilt resistant cultivar FL-EP™‘113’; *P* = *P*-value

## Discussion

### Spotted wilt phenotyping

In this experiment, we evaluated the severity of spotted wilt disease in cultivated peanut using both visual rating and ELISA testing under natural TSWV inoculation potential in a field setting. Mechanical inoculation of TSWV was previously reported [34] and the method of inoculation was successfully applied to cultivated peanut after it was proved to be useful in tobacco (*Nicotiana tabacum*). The inoculation of TSWV in peanut via mechanical inoculation resulted in a transmission rate of approximately 75 to 100% [34]. Although the transmission rate was high in mechanical inoculation, application of this method was still limited in research related with spotted wilt resistance in cultivated peanut due to a variety of factors. One limitation is the labor-intensive and time-consuming nature of the technique for large scale screening. Another limitation is that the mechanism behind spotted wilt field resistance in cultivated peanut is still unknown, but may be related to a modification of the plant’s response to infection, thus may differ significantly from the mechanically inoculated plants. For example, in a previous study, both spotted wilt resistant and susceptible cultivars based on field performance were infected utilizing TSWV mechanical inoculation, and the incidence of infection were similar [35]. Currently, no mechanical inoculation has been tested on the two parental cultivars in this study. It is still unknown if mechanical inoculation can distinguish the spotted wilt resistant cultivar Florida-EP™‘113’ and the spotted wilt susceptible cultivar Georgia Valencia, which needs to be tested in future studies.

Natural inoculation heavily depends on the disease activity in nature and results can vary dramatically under different levels of disease pressure occurring among years and locations. It is inevitable that some plants escape from feeding by thrips during natural inoculation, which makes it difficult to distinguish truly resistant plants from susceptible plants that escaped inoculation, and or from asymptomatic plants. In the F_6_ generation evaluated in this study, 194 individual susceptible plants were selected based on phenotypic expression in the field. One of the reasons for selecting symptomatic plants was to avoid biased phenotyping caused by including individuals into resistance group that are asymptomatic or escaped from inoculation. The other important reason for selecting susceptible plant was the assumption that they would be fixed for the susceptible allele at the QTL locus and the recombinants could be quickly identified based on the phenotype to help narrow down the QTL interval. Spotted wilt disease phenotyping under natural inoculation conditions is usually not reliable when evaluating single plants alone, thus in practice, multiple plants with the same genotypes were typically tested and the average of their phenotype was used as the phenotype score of a specific genotype [20]. Therefore, the current study utilized a whole F_7_ plot derived from each susceptible plant selected in F_6_ for evaluation and the percentage of infected plants in the whole plot was used as the final phenotyping result.

Visual rating was based on the researchers’ experiences and can be subjective in practice. Alternatively, the ELISA test was standardized and was used as a quantitative way to measure the severity of infection, which is expected to be more precise than visual rating. Root crown samples were used in the ELISA test because the root crown is usually the tissue type that is most consistent in indicating TSWV infection [9, 36]. The ELISA test was able to detect the virus in asymptomatic plants, which otherwise could be scored as resistant in the visual rating. In this study, a much lower average score was observed for visual rating than for ELISA indicating the presence of a large number of asymptomatic, infected plants. Florida-EP™ ^‘^113′ was derived from a cross between NC94022 and ANorden [22], and NC94022 was reported to have a high resistance to spotted wilt [7]. None of the 16 roots from Florida-EP™ ^‘^113′ showed positive results from ELISA, which validated the high TSWV resistance of this newly released cultivar.

### QTL analysis

QTL analysis based on ELISA data resulted in a more significant QTL in the targeting region than that based on visual rating, since ELISA can better separate resistant and susceptible plants. The PVE (22.8%) of the QTL detected in this study was very similar to the PVE (22.7%) of the previously reported QTL [20]. The QTL interval detected in the F_6_ population in this study spanned from AHGS 3363 (at 42.6 Mb position of A01 chromosome) to AHGS 1646 (at 43.4 Mb position of A01 chromosome, which was narrower than the range previously reported from AHGS 1646 (43.4 Mb) to AHGS 672 (72.2 Mb). The confidence interval of these two QTLs was adjacent but not overlapping. There are a few possible reasons that might account for the QTL shift. First, the previous linkage map was constructed using an F_2_ population and the current linkage map was constructed based on the F_6_ population. As a result of selecting heterozygous F_5_ individuals at the target QTL region for F_6_ population development in this study, all the other QTLs related with TSWV resistance located in the whole genome should be mostly fixed except the target region on the A01 chromosome. While in the F_2_ population, a large proportion of heterozygous genotypes still existed across the whole genome and the interactions between different QTLs were presumably very different from the interactions in the F_6_ generation. This might be the main reason for the QTL shift. The other reason may be related to the environmental variation and interaction between genotype and environment (G*E). Disease pressure varies in different years and different locations, and genotypes could perform differently at different environments.

A few plants displayed a susceptible phenotype even though the flanking markers of the target QTL on the A01 chromosome showed the resistant genotype. This might be due to the interactions of multiple TSWV-related QTLs and potential epistasis effects, or these few plants could be double recombinants in the QTL region. Based on the average ELISA results from resistant, susceptible and heterozygous genotype groups, we believed that the resistance was dominant at the QTL, which further validated our strategy in selecting the susceptible plants in the F_6_ population for effective mapping of the region controlling TSWV resistance. It was reported that disease resistance in plants is usually determined by dominant genes, although not in every case [37].

### Amplicon-seq

Amplicon-seq of the QTL interval allowed us to develop additional SNP and InDel markers. Two InDel markers were integrated into the QTL mapping, which helped narrow the interval to a 0.8 Mb region. An additional 36 SNP markers were developed, which would be a great marker source for further fine-mapping the QTL interval. Based on the SNPs genotyping results from the eight samples, no informative additional recombination events were detected. It is likely that either the target interval was not fully covered by the amplicon-seq method, or the samples size was not large enough. Fine mapping of the QTL requires genotyping a large population with the SNPs identified in this study.

### TSWV resistance in the US peanut mini-core germplasm

Within the US peanut mini-core germplasm, large differences in spotted wilt resistance, including both visual and ELISA ratings, were observed. However, the major QTL identified in the population derived from the cross between Georgia Valencia and Florida-EP™‘113’ did not explain the variation in TSWV resistance, which indicated that the resistance allelic region at this major QTL was likely to originate from a unique genetic resource, not detectable or represented within the mini core collection. The spotted wilt resistant parental line Florida-EP™‘113’ was derived from a cross between NC94022 and ANorden [24] by the UF Peanut Breeding Program. The NC94022 was created from a cross between N91026E and PI 576638 [7]. The PI 576638, a *hirsuta* type line originated in the highlands of Mexico, has been utilized as a good source of spotted wilt resistance in peanut breeding for over 10 years [7, 38]. The resistance allelic region at the major QTL identified in this study was most likely contributed by PI 576638, which is not included in the mini core collection. Alternatively, the mini core collection contained some accessions showing a very low infection rate among all three mini-core datasets, which can be explored as an additional new genetic resource for spotted wilt resistance in peanut breeding programs. Some of those accessions are PI 493938, PI 356004, PI 337293, PI 493880, PI 476636, PI 461427 and PI 493693, among which PI 356004, PI 493880, PI 493693 are *fastigiata* type while the rest are *hypogaea* type.

## Conclusions

In order to refine the QTL related to spotted wilt resistance, an F_6_ population with a heterozygous QTL interval on the A01 chromosome was genotyped and phenotyped. The QTL analysis indicated a shift of the QTL into a new and relatively small interval of 0.8 Mb between markers AHGS 3363 (42.6 Mb) and AHGS 1646 (43.4 Mb) compared to the previous QTL that was based on an F_2_ population. The new interval spanned 6.56 cM on the linkage map and 0.8 Mb on the physical map, with an LOD score of 8.87 and PVE of 22.8%. Amplicon seq of the refined interval discovered additional markers for future fine-mapping within the QTL. The two flanking markers of the QTL were used to genotype 107 accessions in the US peanut mini-core germplasm in order to evaluate the contributions of the resistance allelic region at the QTL identified. No statistically significant differences were observed between the phenotypes of resistant and susceptible genotype groups in the QTL region. The resistance allelic region at the major QTL controlling TSWV resistance in this study most likely does not exist in the US peanut mini core gene pool, thus the resistance allelic region at the QTL we identified is potentially a unique TSWV resistance source.

## Additional file

Additional file 1: Genotyping data of the bi-parental population derived from Georgia Valencia and Florida-EP™‘113’using SSR and InDel markers. (XLSX 25 kb)

## Abbreviations

cM: centi morgan
DNA: deoxyribonucleic acid
InDel: insertion or deletion
LOD: log of odds
MAS: marker assisted selection
Mb: megabase
NFREC: North Florida Research and Education Center
NGS: next generation sequencing
PAGE: polyacrylamide gel electrophoresis
PIC: polymorphism information content
PSREU: Plant Science Research and Education Unit
PVE: phenotypic variation explained
QTL: quantitative trait loci
RIL: recombinant inbred line
SNP: single nucleotide polymorphism
SSR: simple sequence repeat
TSWV: tomato spotted wilt virus
